# The Effects of General System Justification on Corruption Perception and Intent

**DOI:** 10.3389/fpsyg.2016.01107

**Published:** 2016-07-26

**Authors:** Xuyun Tan, Li Liu, Zhenwei Huang, Wenwen Zheng, Yuan Liang

**Affiliations:** ^1^Beijing Key Lab of Applied Experimental Psychology, School of Psychology, Beijing Normal UniversityBeijing, China; ^2^Institute of Sociology, Chinese Academy of Social SciencesBeijing, China

**Keywords:** general system justification, corruption perception, corrupt intention, institutional trust, system justification theory

## Abstract

Previous research stresses that system justifying belief can weaken corruption perception, by this possibly fostering unjust behaviors. However, general results of the effect of general system justification on corruption are ambiguous, indicating also a lessening impact. We conducted a line of studies trying to elucidate these circumstances by testing the effect of general system justification on corruption perception and intention. In addition, we explored institutional trust as a possible mediator in this process. For this purpose, we conducted three studies. The first two studies examined the association between general system justification and corruption. In Study 1, a correlational design was run using questionnaires to assess the relation between general system justification and corruption perception as well as corruption intention. In Study 2, an experimental design was conducted manipulating general system justification via exposure to high or low system threat condition, then measuring its effect on corruption perception and corrupt intention. In Study 3, two sub-studies using correlational and experimental designs were run to explore the mediating role of institutional trust, respectively. Results replicated former studies showing that general system justification is negatively associated with corruption perception. However, they also showed a negative correlation with corrupt intention. Furthermore, they showed that institutional trust mediated the relation between general system justification and corruption. We suggest to consider these findings to further elucidate the psychological basis underlying different effects of general system justification on human behaviors.

## Introduction

In recent years, an increasing number of studies have explored the causes and consequences of corruption. While economists and sociologists have systematically investigated the relation between corruption and political economic development at a macro level (Bandalos and Finney, 2001; Aidt, 2009; Pellegrini, 2011), psychologists have focused on the influencing factors of corruption at an individual level. For example, collectivism is considered as a propelling force in the endorsement of corruption (Mazar and Aggarwal, 2011; Huang et al., 2015). Previous research has also indicated that some conservative ideologies, such as social dominance orientation, right-wing authoritarianism, and hierarchical ideology, can bolster the propensity for corruption (Bac, 1996; Rosenblatt, 2012; Tan et al., 2016b). However, it remains unknown whether the motivation to maintain the status quo of society affects corruption. If so, what is the psychological process that underlies this relationship? Inspired by system justification theory (Jost and Banaji, 1994; Jost and van der Toorn, 2012) and research on institutional trust, we postulate that general system justification can lessen rather than reinforce corruption, and that institutional trust mediates the relation. The aim of the present study is to test these assumptions.

### General System Justification and Corruption

General system justification refers to the motivation to consciously or unconsciously sustain and provide ideological supports for the stability of society, even when vested interests are harmed (Kay and Jost, 2003; Kay et al., 2005). General system justification has been connected to both negative and positive effects on society. General system justification can satisfy epistemic needs to establish order, structure and certainty, satisfy existential needs to perceive a safe and non-threatening environment, and satisfy relational needs to achieve a “shared reality” with others (Jost et al., 2008a). It is positively associated with a wide range of existential benefits, including life satisfaction and a subjective sense of security, meaning, and mastery (Rankin et al., 2009). Meanwhile, it motivates people to endorse complementary gender stereotypes (Kay and Jost, 2003; Jost and Kay, 2005), and to perceive current unequal salary distributions as more fair and satisfying than equal ones (van der Toorn et al., 2010). General system justification conflicts with ego and group justification motives of disadvantaged groups, which is negatively associated with self-esteem, in-group favoritism, and long-term psychological wellbeing (Jost and van der Toorn, 2012; Kuang and Liu, 2012).

Recent studies have shown that general system justification can also help interpret social issues that are closely related to systems other than those concerned with intergroup relations. Feygina et al. (2010) find that general system justification is positively associated with a greater denial of environmental realities and less commitment to pro-environmental action. Whereas, Vainio et al. (2014) indicate that general system justification is negatively associated with the perceived climate risks affecting an individual’s own food system. Furthermore, Cichocka and Jost (2014) reveal that general system justification is positively associated with some forms of non-disruptive participation. Overall, people with high levels of general system justification express low sensitivity to threatening issues and resist social change and related causal events (e.g., social injustice and harmful systemic phenomena) to sustain the status quo. We assume that general system justification may palliate sensitivity to and intentions of corruption when corruption is understood to pose a threat to society.

Corruption is commonly defined as the misuse of public power for private gains (Treisman, 2000; Ko and Weng, 2011). Because corruption is intentionally hidden, it is almost impossible to interpret and measure directly. Instead, corruption perception and corrupt intention are two often used and important proxies for corruption (Serra and Wantchekon, 2012). Corruption perception refers to people’s subjective views on and assessment of the nature and extent of corruption (Lambsdorff, 2006). Corrupt intention refers to the willingness and propensity to use one’s position or power for perceived personal or in-group gain (Rabl, 2011; Sardžoska and Tang, 2012). In the present research, we focus on these two proxies, and attempt to explore the effect of general system justification on corruption perception and corrupt intention.

General system justification may cause people to deny widespread corruption and to perceive corruption as a one-off case involving just a few people. General system justification dampens the sensitivities to the pervasiveness of social injustice (Feygina et al., 2010; Vainio et al., 2014). Previous research illuminates that, in contrast to social change, people who justify a racially stratified society are often inclined to deny the existence of racism (Jost et al., 2009; Costa-Lopes et al., 2013; França and Monteiro, 2013). Other studies also indicate that people with high general system justification manifest a strong denial of environmental realities and a reluctance to bear personal responsibility, as acknowledging environmental problems and adopting environmental protections would threaten the foundation of the social status quo (Feygina et al., 2010; Vainio et al., 2014). Similarly, a heightened perception of corruption may also create structural reforms, challenge ways of life and threaten the status quo. The more people are motivated to bolster the existing system, the more likely they are to perceive less corruption. Thus, we hypothesize that general system justification is negatively associated with corruption perception (Hypothesis 1).

As previously mentioned, previous research indicates that general system justification is not only associated with the subjective perception of conservative issues, but also positively associated with the intention to engage in conservative behaviors (e.g., compensatory stereotype), and is negatively associated with the support of corresponding reforms (e.g., environmental and egalitarian reforms; Kay and Jost, 2003; Jost et al., 2004; Kay et al., 2005; Feygina et al., 2010). However, what about other unjust behaviors that threaten and challenge society, such as corruption? According to system justification theory, neglecting and rejecting them may be an effective way to avoid system threats and to bolster the stability and legitimacy of the status quo (Hennes et al., 2012; Jost and van der Toorn, 2012). Thus, when confronted with such behaviors, general system justification may be activated and dampen the intention to indulge in such behaviors. Corruption is not only a platform to display the privilege and preserve the disproportionate benefits of dominants, but also a behavior that generally harms both national stability and global security and threatens the general society (Wei, 2000; Aguilera and Vadera, 2008; Rotberg, 2009). Contrary to people devoted to sustaining the vested interest for themselves or their in-groups, when confronted with a choice of either engaging in corruption or avoiding possible threats toward society, individuals with high general system justification are motivated to inhibit their corrupt intention and to resist engaging in corruption. Therefore, we hypothesize that general system justification is negatively associated with corrupt intention (Hypothesis 2).

### Institutional Trust as a Mediator

Institutional trust refers to the degree of satisfaction with and confidence in public institutions (Mishler and Rose, 2001; Chang and Chu, 2006; Morris and Klesner, 2010). Previous research demonstrates that institutional trust enhances political involvement and the effectiveness of governments and institutions (Mishler and Rose, 2005; Rus and Iglič, 2005), and it plays an essential role in retaining online inter-organizational relationships (Pavlou et al., 2003). It also positively affects citizens’ well-being (Hudson, 2006), confidence (Metlay, 1999), satisfaction with democracy, and happiness (Ekici and Koydemir, 2013).

General system justification can activate high institutional trust to deny potential risks. Previous research indicates that general system justification correlates with the existential benefits of increasing one’s life satisfaction, sense of security, and trust in the existing system (Jost et al., 2003; Rankin et al., 2009). Vainio et al. (2014) indicate that general system justification enhances institutional trust, then reduces the perception of food risks and resists personal activities that threaten the existing food system. Jost et al. (2004) point out that general system justification against threats of terrorist attacks increases trust in the government. It might be possible that institutional trust is not just a consequence of general system justification, but it also plays an active role in the perception of, and the intention to engage in, corruption.

Much of the recent research identifies institutional trust as a critical cause of corruption. Previous research argues that lower institutional trust leads to increased corruption (Mishler and Rose, 2001; Chang and Chu, 2006). Xin and Rudel (2004) contend that an atmosphere of low institutional trust increases the levels of corruption perception, and thereby provides a justification for such behavior. Morris and Klesner (2010) also indicate that the level of institutional trust is negatively associated with corruption perception and participation in corruption. Lack of trust in the government prevents the adoption of ethos-based behavior and instead favors instrumental approaches to problems instead. Distrust in institutions thus fosters a tolerant attitude toward corruption, activates a widespread perception of corruption, and feeds individual participation in such acts as well as corrupt intention (Morris and Klesner, 2010). In sum, general system justification motivates people to trust in the institutions, which in turn palliates their sensitivity to corruption and inhibits their corrupt intention. Therefore, we hypothesize that institutional trust mediates the lessening effects of general system justification on corruption perception and corrupt intention (Hypothesis 3).

### Overview of Present Research

We ran three studies to test our hypotheses: (1) general system justification is negatively associated with corruption perception and corrupt intention, and (2) institutional trust mediates these relationships. Study 1 aimed to examine the correlations between general system justification and perception and intention of corruption. General system justification was measured via perception levels regarding whether general Chinese society is fair, legitimate, and justifiable. A questionnaire and a corrupt scenario were used to measure the perception of and intention to engage in corruption, respectively. In Study 2, an experiment was conducted to further investigate general system justification and its associations with both corruption perception and corrupt intention. An experimental manipulation design was employed to increase general system justification, and to observe its subsequent effects on corruption perception and corrupt intention, which were measured by a different questionnaire and scenario. In Study 3, two sub-studies using correlational and experimental designs, respectively, were conducted to explore the mediating role of institutional trust in the relationship between general system justification and corruption perception as well as corrupt intention. All studies have been reviewed and approved by the Committee of Protection of Subjects at Beijing Normal University.

## Study 1: Correlational Relationship Between General System Justification and Corruption

### Materials and Methods

#### Participants

In total, 457 individuals (213 women and 244 men) voluntarily participated in this study. Most participants were college students recruited from two universities in Shandong Province in China. The remainder were recruited from a continuing education class in a university in Shandong Province in China, including middle school teachers, managers, and other professionals. The mean age of the sample was 21.25 years old (*SD* = 1.806). Among the participants, 76.0% evaluated their economic position as fair, 7.9% as rich, and 16.1% as poor; 36.9% came from cities, and 63.1% came from rural areas.

#### Materials

##### General system justification

An eight-item measure adapted from a general system justification scale (Kay and Jost, 2003) was used to measure participants’ general system justification. For example, one item was “In general, China is just and fair.” The full scale is listed in Appendix A. Participants were instructed to indicate their agreement with each statement on a nine-point Likert scale, from 1 (“completely disagree”) to 9 (“completely agree”). The original English scale items were translated into Chinese and back translated for accuracy by two independent teams that were blinded to the research hypotheses. The average score of these eight items was calculated as a general system justification index, with higher ratings representing stronger general system justification (Cronbach’s α = 0.727).

##### Corruption perception

To capture participants’ corruption perception, a five-item corruption perception measure (Tan et al., 2016a) was used. For example, one item was “The problem of corruption is very severe in Chinese society today.” The full scale is listed in Appendix B. Participants were instructed to indicate their agreement with each statement on a nine-point Likert scale, from 1 (“completely disagree”) to 9 (“completely agree”). The average score of these five items was calculated as a corruption perception index, with higher ratings representing a higher corruption perception (Cronbach’s α = 0.725).

##### Corrupt intention

A corrupt scenario (Li et al., 2006; Tan et al., 2016a) was used to measure participants’ corrupt intention. After reading the scenario, participants were asked to answer four questions about corrupt intention. The scenario and questions are shown in Appendix C. The responses to the items were provided on a nine-point Likert scale, from 1 (“completely disagree”) to 9 (“completely agree”). The average score of these four items was calculated as a corrupt intention index, with higher ratings representing higher corrupt intention (Cronbach’s α = 0.710).

#### Procedure

After providing informed consent, participants were instructed to complete questionnaires in their classrooms; the questionnaires included items relating to the general system justification scale, the corruption perception measure, and the corrupt intention measure (based on the corrupt scenario). Some unrelated measures, such as self-construal and moral outrage, were also included in the questionnaires to keep participants from guessing the purpose of the study. All participants viewed the sections and items in the same order. Participants completed the questionnaires in groups of 40–50 per classroom, within a period of half an hour. After completing all measures, participants were asked to provide demographic information. Each participant received a gift costing approximately ¥10 upon the completing of the survey for their participation and effort.

### Results and Discussion

The descriptive statistics for general system justification, corruption perception, and corrupt intention can be seen in **Table 1**

**Table 1:** Correlation matrix, means, and standard deviations for all variables in Study 1.

	Mean	*SD*	2	3	4	5
1. General system justification	4.94	1.294	-0.172^∗∗^	-0.168^∗∗^	-0.004	-0.054
2. Corruption perception	7.46	1.284		-0.034	-0.082	0.023
3. Corrupt intention	3.86	1.632			-0.084	0.131^∗∗^
4. Gender	–	–				–
5. Age	21.25	1.806				

*^∗∗^*p* < 0.01.*

We performed linear regression analyses to examine our hypotheses, adjusting for sex, age, birthplace, and economic status in each model. As expected, general system justification negatively predicted corruption perception [β = -0.174, *t*(443) = -3.701, *p* < 0.001] and corrupt intention [β = -0.167, *t*(443) = -3.573, *p* < 0.001]. These analyses indicated that participants with higher general system justification perceived less corruption and manifested lower corrupt intention. Thus, Hypotheses 1 and 2 are supported.

## Study 2: The Effects of General System Justification on Corruption

Study 1 examined general system justification and its association with corruption perception and corrupt intention. To further examine the lessening effects of general system justification on corruption, in Study 2 we ran an experiment to manipulate general system justification and used it to investigate the palliate effects. Furthermore, to avoid the possible confounds of *guanxi^1^* and corruption in the specific scenario used in Study 1, another questionnaire was employed to measure corrupt intention. Kay et al. (2005) report that people generate a high general system justification when confronted with strong challenges and threats to the existing system, and vice versa. Following their research paradigm, we manipulated participants’ general system justification by exposure to high/low system-threatened conditions and then examined the subsequent effects on corruption perception and corrupt intention.

### Materials and Methods

#### Participants

In total, 73 individuals (61 women and 12 men) were recruited from a university in Shandong Province in China, they voluntarily participated in the study. The mean age of the sample was 21.78 years old (*SD* = 0.854). Among participants, 89.0% evaluated their subjective economic status as fair, with 16.4% coming from large and medium-sized cities, 46.6% from county-level cities, and 37% came from rural areas. The participants were randomly assigned to the high general system justification condition (*n* = 37) or the low general system justification condition (*n* = 36).

#### Materials

##### Manipulation of general system justification

Following the paradigm used by Kay et al. (2005) to manipulate general system justification beliefs, participants were asked to read one of two short passages apparently written by a local journalist. Those assigned to the high general system justification condition were provided a passage reporting that political, social, and economic situations in China were worsening, which threatened participants’ beliefs in the justification of the current general society. In contrast, those assigned to the low general system justification condition, were provided a passage reporting that China’s political, social and economic situations were relatively positive and stable. The specific passages for the two conditions are shown in Appendix D.

After reading the passage, participants completed recall tests and the adapted general system justification scale (Kay and Jost, 2003) for a manipulation check.

##### Corruption perception

Seven items concerning corruption perception selected from the World Value Survey (Inglehart, 2000) and General Social Survey (Davis and Smith, 1991) were used to measure participants’ corruption perception. For example, one item was “How widespread do you think corruption is in the public service in China?” The full scale is listed in Appendix E. Participants were instructed to indicate their corruption perception on a nine-point Likert scale, from 1 to 9. The average score of these seven items was calculated as a corruption perception index, with higher ratings representing stronger corruption perception (Cronbach’s α = 0.721).

##### Corrupt intention

A 14-item corrupt intention scale (Leong and Lin, 2009) was used to measure participants’ corrupt intention. For example, one item was “Although everyone in my organization exhibits corrupt behaviors, I still would not act in that way.” The full scale is listed in Appendix F. Participants were instructed to indicate their agreement with each statement on a nine-point Likert scale, from 1 (“completely disagree”) to 9 (“completely agree”). The average score of these 14 items was calculated as a corrupt intention index, with higher ratings representing stronger corrupt intention (Cronbach’s α = 0.765).

#### Procedure

After arriving at the laboratory, participants provided their informed consent. They were then informed that they were going to participate in several different studies, whereby the first study concerned a two-stage memory task, which was actually a cover story. They were asked to carefully read a passage, and then to recall information about this story later in the session. After completing the recall task in the first stage, they were told that because it was important to let time pass before they could complete the recall task in the second stage (to allow for memory decay), they would work on another survey regarding attitudes toward social life. After completing the survey, participants were asked to complete the second-stage recall task. At the end of the tasks, a debriefing procedure was conducted (Bargh, 2000). Participants were asked what they thought the purpose of the experiment was, whether the different tasks were related, and whether their behavior in one task was influenced by what they did in another. Participants mentioned no association between the memory task and the social attitude survey. Finally, participants were paid ¥10 in cash for their participation and effort.

### Results and Discussion

First, a *post hoc* power analysis was conducted to determine whether the study was of an adequate sample size to perform the planned statistical analyses. Achieved powers were more than 85.7% for the perception and intention of corruption with the sample size of 73 in addition to using a two-sided statistic and an alpha level of 0.05. This demonstrated that the sample size was large enough to address the specific aims of Study 2.

To examine the effectiveness of the manipulation, an independent sample *t*-test was performed using the manipulating condition (high vs. low general system justification) as the independent variable and general system justification as the dependent variable. Our results confirmed that the manipulation had a significant effect [*t*(71) = 2.464, *p* = 0.016, and *Cohen’s d* = 0.569]. Participants exposed to the high general system justification condition (*M* = 5.74, *SD* = 1.242) had higher general system justification scores than those exposed to the low general system justification condition (*M* = 5.10, *SD* = 0.950). In addition, the results showed that both sex and age were not significant (*p*s > 0.05).

To test the effects of general system justification on corruption perception and corrupt intention, we performed two independent sample *t*-tests. The results indicated that participants exposed to the high general system justification condition had lower corruption perception scores (*M* = 5.13, *SD* = 1.116) than those exposed to the low general system justification condition [*M* = 5.89, *SD* = 1.030; *t*(71) = -3.027, *p* = 0.003, and *Cohen’s d* = -0.699]. Participants exposed to the high general system justification condition had lower corrupt intention scores (*M* = 3.96, *SD* = 1.056) than those exposed to the low general system justification condition [*M* = 4.70, *SD* = 0.562; *t*(71) = -3.765, *p* < 0.001, and *Cohen’s d* = -0.870]. Hence, Hypotheses 1 and 2 were also supported.

## Study 3: Institutional Trust as a Mediator

Both Studies 1 and 2 support Hypotheses 1 and 2; that is high general system justification is associated with low corruption perception and low corrupt intention. In Study 3, we intended to further explore the psychological processes related to the lessening effect of general system justification on corruption, and thereby to test the hypothesis that institutional trust mediates the relationship. Two sub-studies were conducted. Sub-study 3.1 used a correlational design to explore the mediating role of institutional trust in the relation between general system justification and corruption. An experimental design was employed in Sub-study 3.2 to further examine the mediating mechanism of institutional trust in the effect of general system justification on corruption.

### Materials and Methods

#### Participants

In Sub-study 3.1, 368 participants (89 women and 279 men) were recruited from three universities in Shandong, Hebei, and Hunan Provinces in China, and they voluntarily participated in the study. The mean age of the sample was 19.63 years old (*SD* = 2.942). Among participants, 69.9% evaluated their subjective economic status as fair, 20.5% as rich, and 9.6% as poor. Additionally, 23.1% came from large and medium-sized cities, 16.8% from county-level cities, and 60.1% from rural areas.

In Sub-study 3.2, 112 participants (60 women, 52 men) were recruited online via a smartphone app called *Wenjuanbao*. *Wenjuanbao* is a Chinese professional online survey and market research website that has over one million members. The mean age of the sample was 28.41 years old (*SD* = 7.80). Participants were randomly assigned to either the high general system justification condition (*n* = 56) or the low general system justification condition (*n* = 56).

#### Materials

##### General system justification

In Sub-study 3.1, we administered the same general system justification scale as used in Study 1 to measure participants’ general system justification (Cronbach’s α = 0.785).

##### Manipulation of general system justification

In Sub-study 3.2, the same paradigm employed in Study 2 was used to manipulate general system justification.

##### Institutional trust

To gage the level of institutional trust, participants were presented with 17 institutions (e.g., the Supreme Court, local government, television, and hospitals) and asked to indicate the level of trust they have in each of them. The responses to these items were provided on a nine-point Likert scale, from 1 (“completely distrust”) to 9 (“completely trust”). The average score of these 17 items were calculated as an institutional trust index, with higher ratings representing greater institutional trust in Sub-study 3.1 (Cronbach’s α = 0.952) and Sub-study 3.2 (Cronbach’s α = 0.949).

##### Corruption perception

The same corruption perception scale as that used as in Study 2 was used to measure participants’ corruption perception in Sub-study 3.1 (Cronbach’s α = 0.784) and Sub-study 3.2 (Cronbach’s α = 0.714).

##### Corrupt intention

The same corrupt intention scale used as in Study 2 was used to measure participants’ corrupt intention in Sub-study 3.1 (Cronbach’s α = 0.766) and Sub-study 3.2 (Cronbach’s α = 0.744).

#### Procedure

The procedure used in Sub-study 3.1 resembled that used in Study 1 but for the addition of the institutional trust measure. The same paradigm used in Study 2 was employed in Sub-study 3.2 to manipulate general system justification and observe the mediating mechanism of institutional trust underlying the causal effect. Each participant was randomly provided with one of two links on a smartphone app, representing the two experiment manipulations: high or low general system justification. At the end of either Sub-study, each participant received ¥ 10 in cash or a gift costing approximately ¥ 10 upon completing the survey for their participation and effort.

### Results and Discussion

#### Preliminary Analysis

The descriptive statistics of all variables measured in Sub-study 3.1 can be seen in **Table 2** Sex and age were significantly associated with most variables. Thus, we included the covariance paths for age and sex in our mediation analyses.

**Table 2:** Correlation matrix, means, and standard deviations for all variables in Study 3.1.

	Mean	*SD*	2	3	4	5	6
1. General system justification	5.34	1.410	0.534^∗∗^	-0.449^∗∗^	-0.299^∗∗^	-0.227^∗∗^	-0.288^∗∗^
2. Institutional trust	6.26	1.571		-0.373^∗∗^	-0.322^∗∗^	-0.245^∗∗^	-0.333^∗∗^
3. Corruption perception	5.37	1.377			0.298^∗∗^	0.367^∗∗^	0.376^∗∗^
4. Corrupt intention	4.21	1.095				0.112^∗^	0.224^∗∗^
5. Sex	-	-					-
6. Age	19.63	2.962					

*^∗^*p* < 0.05, ^∗∗^*p* < 0.01.*

To examine the effectiveness of the manipulation in Sub-study 3.2, an independent sample *t*-test was performed using the manipulating condition (high vs. low general system justification condition) as the independent variable and general system justification as the dependent variable. Our results confirmed that the manipulation had a significant effect [*t*(110) = -3.053, *p* = 0.003, and *Cohen’s d* = -0.577]. Participants assigned to the high general system justification condition (*M* = 5.40, *SD* = 1.306) had higher general system justification scores than those assigned to the low general system justification condition (*M* = 4.70, *SD* = 1.124). The mean and standard deviation of institutional trust, corruption perception, and corrupt intention in the two general system justification conditions can be seen in **Table 3**

**Table 3:** The mean and standard deviation of institutional trust, and perception of and intention to engage in corruption in different general system justification conditions in Study 3.2.

	Institutional trust		Corruption perception		Corrupt intention	
	Mean	*SD*	Mean	*SD*	Mean	*SD*
High general system justification condition	5.51	1.095	5.90	1.223	4.75	1.096
Low general system justification condition	6.06	1.426	5.45	1.096	4.05	1.138

#### Mediation Analysis of Sub-study 3.1

**FIGURE 1:**
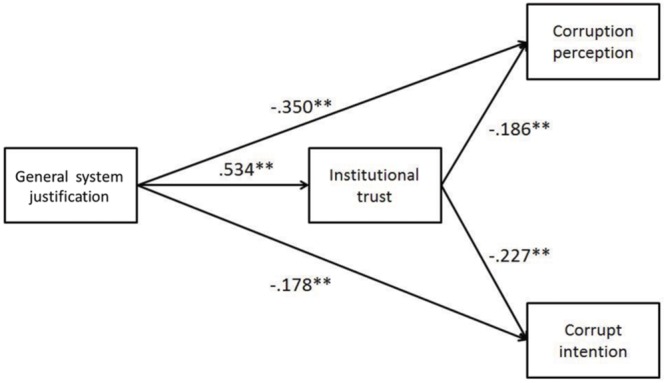
**Hypothesized path model of the relationships between system justification, institutional trust, corruption perception, and corrupt intention in Study 3.1.** ^∗∗^*p* < 0.01. Path coefficients are standardized. Significant paths are denoted using solid lines.

A confirmatory factor analysis was employed to examine all items of general system justification and institutional trust measures. The results indicate that general system justification and institutional trust are two related but separate constructs (*r* = 0.456, *p* < 0.001, χ^2^/*df* = 2.788, *IFI* = 0.964, *CFI* = 0.963, and *RMSEA* = 0.070). We built a model (as in **Figure 1**) using AMOS 18.0 and used maximum likelihood estimation to examine our hypothesis that institutional trust mediates the impact of general system justification on corruption perception and corrupt intention. The analysis results indicated an acceptable model fit *(*χ^2^/*df =* 2.741, *df =* 2, *p* = 0.064, *GFI* = 0.995, *NFI =* 0.989, *IFI =* 0.993, *CFI =* 0.993, and *RMSEA =* 0.069).

As shown in **Figure 1**, general system justification was positively correlated with institutional trust [β = 0.534, *t*(365) = 12.094, *p* < 0.001]. General system justification negatively predicted corruption perception [β = -0.350, *t*(365) = -6.458, *p* < 0.001] and institutional trust negatively predicted corruption perception [β = -0.163, *t*(365) = -3.432, *p* < 0.001]. General system justification was negatively predicted corrupt intention, [β = -0.178, *t*(365) = -3.085, *p* = 0.002] and institutional trust negatively predicted corrupt intention [β = -0.227, *t*(366) = -3,931, *p* < 0.001]. Thus, the mediation hypothesis was also supported.

To further prove the mediation hypothesis, we also employed a bootstrapping technique with a bootstrap resample (*n* = 5000; Preacher and Hayes, 2008). The results showed that the 95% confidence interval for the indirect effect of general system justification on corruption perception was [-0.448, -0.230], and the 95% confidence interval for the indirect effect of general system justification on corrupt intention was [-0.169, -0.030], neither of which included zero. The 95% confidence interval for the direct effect of general system justification on corruption perception was [-0.238, -0.035], and the 95% confidence interval for the direct effect of general system justification on corrupt intention was [-0.168, -0.037]. Thus, these results supported Hypothesis 3. Specifically, institutional trust mediates the correlations between general system justification and corruption perception, and between general system justification and corrupt intention.

#### Mediation Analysis of Sub-study 3.2

**FIGURE 2:**
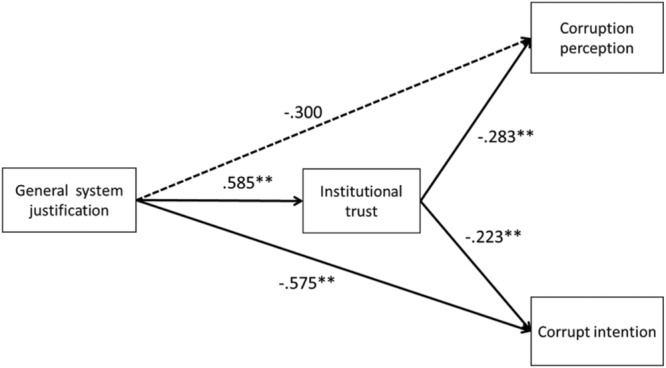
**Hypothesized path model of the relationships between general system justification, institutional trust, corruption perception, and corrupt intention in Study 3.2.** ^∗∗^*p* < 0.01. Path coefficients are standardized. Significant paths are denoted using solid lines. Non-significant paths are denoted using dashed lines.

The results showed that sex was not significantly associated with all observed variables (*p*s > 0.294), and age was significantly associated with general system justification (*r* = -0.231, *p* = 0.014) and institutional trust (*r* = -0.278, *p* = 0.003). Controlling the two variables, we built a model (as shown in **Figure 2**) with AMOS 18.0, and used maximum likelihood estimation to examine our hypothesis that institutional trust mediates the effects of general system justification on corruption perception and corrupt intention. The analysis results indicated a good model fit (χ^2^/*df* = 1.090, *NFI* = 0.946, *IFI* = 0.995, *CFI* = 0.995, and *RMSEA* = 0.028).

As shown in **Figure 2**, when controlling for sex and age, the effect of general system justification on institutional trust was significant [β = 0.575, *t*(110) = 2.512, *p* = 0.012]. The direct effect of general system justification on corruption perception was not significant [β = -0.300, *t*(110) = -1.410, *p* = 0.159]. Even so, the result still showed a consistent trend with the hypothesis. The direct effect of general system justification on corrupt intention was significant [β = -0.585, *t*(110) = -3.451, *p* < 0.005]. The mediation hypothesis was confirmed.

To further examine the mediation hypothesis, we also conducted a bootstrapping technique (Preacher and Hayes, 2008). A total of 5,000 bootstrap samples were used for the estimates. The results showed that the 95% confidence interval for the direct effect of general system justification on corruption perception was [-0.718, 0.097], while that of the indirect effect was [-0.366, -0.009], and did not include zero. The 95% confidence interval for the direct effect of general system justification on corrupt intention was [-0.930, -0.240], and that of the indirect effect was [-0.334, -0.009], both of which did not include zero. These results further confirmed Hypothesis 3; that is, institutional trust mediates the effects of general system justification on corruption perception and corrupt intention.

## General Discussion

Previous research often stresses that conservative ideologies can weaken corruption perception, by this possibly fostering corruption. However, general results of the effects of general system justification on corruption are ambiguous, indicating also a lessening impact. We conducted a line of studies trying to elucidate these circumstances by testing the effect of general system justification not only on corruption perception, but also on corruption intention. Furthermore, we explored institutional trust as a possible mediator in this process. The findings demonstrate that general system justification is negatively associated with corruption perception and corrupt intention. In addition, institutional trust plays a mediating role in these relations.

The present research provides a new perspective to explore the influencing factors of corruption at an individual level. For many years, scholars have conducted substantial research to explore the influencing factors and consequences of corruption from different macro perspectives. More recently, a number of social psychologists have focused on corruption, attempting to explore the psychological factors and mechanisms that underlie the concept. Mazar and Aggarwal (2011) indicate that a sense of responsibility mediates the effect of collectivism on the likelihood of engaging in bribery. Bai et al. (2014) show that perceived punishment is a boundary condition for the positive effects of the belief in a just world to others on corruption. Tan et al. (2016a) point out that social dominance orientation is positively associated with a reduced awareness of corruption by inhibiting moral outrage. To fully reveal the behavioral mechanism, it would be worthwhile to further explore the cause and effect of corruption with a combination of macro and microanalyses.

These findings once again provide direct empirical evidence that general system justification is negatively associated with corruption perception. They add to the growing literature that suggests that system-justifying ideologies including general system justification, reduce individuals’ sense and perception of social injustice; for example, the denial of global warming (Feygina et al., 2010), racism (Jost et al., 2009; Costa-Lopes et al., 2013; França and Monteiro, 2013), and sexism (Jost et al., 2002; Binning and Sherman, 2011). Consistent with these conclusions, our findings indicate that people are disposed to maintain an existing system against threats by minimizing or even denying corruption, preventing the need for political restructuring and systemic changes. In addition, our findings also add to the growing literature suggesting that corruption perception is positively correlated with corrupt intention, but the relationship seems not stable (Treisman, 2007; Tan et al., in press). Such association might indicate that participants estimate their intent on the base of their corruption perception, that is they are only estimating their intention lower as they perceive fewer circumstances as actually corruptive. To really advance measures of corruption reduction future research has thus to disentangle if general system justification has a genuine effect on corruption intent or if this is just mediated by a weakened corruption perception.

We find that general system justification negatively predicts corrupt intention, which suggests that the general system justification theory can not only account for the advocacy and support of unjust behaviors (Ledgerwood et al., 2011; Phelan and Rudman, 2011), but also provides insight into why individuals resist some injustices (e.g., corruption). Feygina et al. (2010) assume that there are at least two types of social injustice that have different relations and functions with the status quo. One includes systemic problems in the existing system such as social hierarchy (Kay et al., 2005), meritocracy (Jost et al., 2008b), and complementary stereotypes (Kay and Jost, 2003). These problems are supported and bolstered to sustain and enhance the existing system. The other manifests itself in threats and challenges to the status quo; for example, terrorist attacks (Bonanno and Jost, 2006) and corruption. Individuals with high general system justification are willing to reject and defeat them to maintain systemic stability and have limited intention to commit such injustices. The relations between general system justification and the two types of social injustice should be further explored in future research.

Previous findings also imply that various conservative ideologies may have different effects on corrupt intention. Some scholars explore the relationships between social conservative ideologies and corruption, and achieve a complex outcome. Previous research demonstrates that group-based conservative ideologies, such as right-wing authoritarianism and meritocratic ideology, are positively associated with corrupt actions (You, 2007; Rosenblatt, 2012; Tan et al., 2016b; Tan et al., in press). As corruption highlights the corrupters’ group-dominant positions, personal power, and prestige (You, 2007), the initiation and maintenance of corruption may be coordinated by the support of group-based hierarchies (Rosenblatt, 2012). Nevertheless, Bai et al. (2014) indicate that belief in a just world to others can reduce perceived intentions of corruptive behaviors. Our research demonstrates that general system justification reduces corrupt intention, to avoid the threats of corruption on the stability of general society. Above all, various conservative ideologies may exert different impacts on the propensity for corruption, which may depend on the beneficial or threatening role of corruption in a system. Therefore, the complicated impacts of conservative ideologies on social behaviors should be explored further in future research.

Consistent with a number of previous studies, our findings demonstrate that general system justification increases institutional trust (Chanley, 2002; Jost et al., 2003), and that institutional trust is negatively associated with corruption perception and corrupt intention (Chang and Chu, 2006; Morris and Klesner, 2010). Our findings further support the mediating role of institutional trust in the relation between general system justification and corruption. To maintain a stable society, individuals perceive institutions as more trustworthy and legitimate, which leads to low corruption perception and a low likelihood of corrupt intention. In addition, the findings are consistent with previous research (Vainio et al., 2014) that general system justification and institutional trust are two related but independent notions. Nevertheless, we are not sure whether general system justification simply causes institutional trust or there is mutual causation between the two. Therefore, it is worthwhile to further examine the causal relationships among general system justification, institutional trust, and corruption.

In practice, when general system justification has negative impacts on corruption perception and corrupt intention, there are significant implications for anti-corruption efforts. Transparency International releases an annual Corruption Perceptions Index (CPI), testing countries’ corruption perception levels with a 100-score scale. The CPI scores of 121 countries/territories (among 175 surveyed) are below 50, which implies that corruption is almost ubiquitous in many countries (Transparency International, 2014). Previous research indicates that besides corruption reality, the subjective perception of corruption itself can also influence investment decisions, economic growth, and political behaviors (Mauro, 1995; Treisman, 2000). The present research implies that general system justification and increased institutional trust can be obtained by highlighting the threat of corruption to society and enhancing the honesty and credibility of existing social institutions. This will act to effectively buffer individuals’ propensity for corruption and eventually decrease corrupt behaviors. In addition, as a lower perception of corruption could also affect the judgment of one’s own unjust and immoral corrupt actions, through other approaches, such as engaging in morally responsible behaviors (Ntayi et al., 2013), we can try to diminish the palliative function of general system justification on corruption perceptions and activate sensitivities to corruption.

The limitations and prospects of the present study should be noted. First, this study only focuses on perceptions of and intentions to engage in corruption with self-report measures. However, social desirability is an inevitable bias of the self-report measure. Second, a further confounding factor is demand characteristics. Previous research indicates that corruption is often seen as highly negative and most people will deny engaging in corrupt behavior. At the same time, when switching the referent to others, corrupt intention increases (Ferreira et al., 2012). Therefore, developing a suitable and pure behavioral measure of actual corruption may effectively solve the above problems, and represents a worthwhile avenue for future research. Third, as most participant groups in the current research had a mean age of approximately 20 years, a wider age range should be employed in the future, as perceptions and intentions of corruption might very well be dependent on employment situations and opportunities for corruption. Fourth, general system justification also interferes with forming an intention to correct injustices (Feygina et al., 2010). In the future, we should pay attention to the ways in which we can overcome the negative impacts of general system justification on anti-corruption reforms. Finally, there may be some cross-cultural/national differences. The different country specificities, like advocating harmony or preferring authority, may lead to different impacts of general system justification on corruption. Thus, it would be worthwhile to investigate the cultural differences influencing the relationship between general system justification and corruption in future.

## Author Contributions

The first author, XT, contributed to all aspects of work for this article. LL contributed to conception, and design and revising the article critically. ZH, WZ, and YL contributed to data analysis and interpretation and revising the article critically.

## Conflict of Interest Statement

The authors declare that the research was conducted in the absence of any commercial or financial relationships that could be construed as a potential conflict of interest.

The reviewer KH and handling Editor declared their shared affiliation, and the handling Editor states that the process nevertheless met the standards of a fair and objective review.

## Footnotes

1 The scenario employed in Study 1 may invoke both corruption and *guanxi* relationships, as the person who gives money is a friend of “mine.” Previous research indicates that participants of corruption are not compelled to engage in such conduct because of *guanxi* (Li, 2011). Therefore, although the scenario seems not to disentangle the personal relationship from the corruption context, it is still an effective way to measure the corrupt intention of the participants.

## References

[B1] Aguilera R. V. Vadera A. K. (2008). The dark side of authority: antecedents, mechanisms, and outcomes of organizational corruption. *J. Bus. Ethics* 77 431–449. 10.1007/s10551-007-9358-8

[B2] Aidt T. S. (2009). Corruption, institutions, and economic development. *Oxf. Rev. Econ. Policy* 25 271–291. 10.1093/oxrep/grp012

[B3] Bac M. (1996). Corruption, supervision, and the structure of hierarchies. *J. Law, Econ. Organ.* 12 277–298.

[B4] Bai B.-Y. Liu X.-X. Kou Y. (2014). Belief in a just world lowers perceived intention of corruption: the mediating role of perceived punishment. *PLoS ONE* 9:e97075 10.1371/journal.pone.0097075 PMC4023923 24835428

[B5] Bandalos D. L. Finney S. J. (2001). “Item parceling issues in structural equation modeling,” in *New Developments and Techniques in Structural Equation Modeling* eds Marcoulides G. A. Schumacker R. E. (Mahwah, NJ: Lawrence Erlbaum Associates, Inc)269–296.

[B6] Bargh J. (2000). “The mind in the middle: A practical guide to priming and automaticity research,” in *Handbook of Research Methods in Social and Personality Psychology* eds Judd H. T. Reis M. C. (New York, NY: Cambridge University Press), 253–285.

[B7] Binning K. R. Sherman D. K. (2011). Categorization and communication in the face of prejudice: When describing perceptions changes what is perceived. *J. Pers. Soc. Psychol.* 101 321–336. 10.1037/a0023153 21463076

[B8] Bonanno G. A. Jost J. T. (2006). Conservative shift among high-exposure survivors of the september 11th terrorist attacks. *Basic Appl. Soc. Psychol.* 28 311–323. 10.1207/s15324834basp2804_4

[B9] Chang E. C. Chu Y. H. (2006). Corruption and trust: exceptionalism in Asian democracies? *J. Polit.* 68 259–271. 10.1111/j.1468-2508.2006.00404.x

[B10] Chanley V. A. (2002). Trust in government in the aftermath of 9/11: determinants and consequences. *Polit. Psychol.* 23 469–483. 10.1111/0162-895X.00294

[B11] Cichocka A. Jost J. T. (2014). Stripped of illusions? Exploring system justification processes in capitalist and post-Communist societies. *Int. J. Psychol.* 49 6–29. 10.1002/ijop.12011 24811719

[B12] Costa-Lopes R. Dovidio J. F. Pereira C. R. Jost J. T. (2013). Social psychological perspectives on the legitimation of social inequality: past, present and future. *Eur. J. Soc. Psychol.* 43 229–237. 10.1002/ejsp.1966

[B13] Davis J. A. Smith T. W. (1991). *The NORC General Social Survey: A User’s Guide*, Vol. 1 London: SAGE.

[B14] Ekici T. Koydemir S. (2013). Social capital, government and democracy satisfaction, and happiness in Turkey: a comparison of surveys in 1999 and 2008. *Soc. Indic. Res.* 118 1031–1053.

[B15] Ferreira M. Fischer R. Porto J. Pilati R. Milfont T. (2012). Unraveling the mystery of brazilian jeitinho: a cultural exploration of social norms. *Pers. Soc. Psychol. Bull.* 38 331–344. 10.1177/0146167211427148 22143307

[B16] Feygina I. Jost J. T. Goldsmith R. E. (2010). System justification, the denial of global warming, and the possibility of “system-sanctioned change”. *Pers. Soc. Psychol. Bull.* 36 326–338. 10.1177/0146167209351435 20008965

[B17] França D. X. Monteiro M. B. (2013). Social norms and the expression of prejudice: the development of aversive racism in childhood. *Eur. J. Soc. Psychol.* 43 263–271. 10.1002/ejsp.1965

[B18] Hennes E. P. Nam H. H. Stern C. Jost J. T. (2012). Not all ideologies are created equal: epistemic, existential, and relational needs predict system-justifying attitudes. *Soc. Cogn.* 30 669–688. 10.1521/soco.2012.30.6.669

[B19] Huang Z.-W. Liu L. Zheng W.-W. Tan X.-Y. Zhao X. (2015). Walking the straight and narrow: the moderating effect of evaluation apprehension on the relationship between collectivism and corruption. *PLoS ONE* 10:e0123859 10.1371/journal.pone.0123859 PMC4376769 25815819

[B20] Hudson J. (2006). Institutional trust and subjective well-being across the EU. *Kyklos* 59 43–62. 10.1111/j.1467-6435.2006.00319.x

[B21] Inglehart R. (2000). *Codebook for World Values Survey.* Ann Arbor, MI: Institute for Social Research.

[B22] Jost J. T. Banaji M. R. (1994). The role of stereotyping in system-justification and the production of false consciousness. *Br. J. Soc. Psychol.* 33 1–27. 10.1111/j.2044-8309.1994.tb01008.x

[B23] Jost J. T. Banaji M. R. Nosek B. A. (2004). A decade of system justification theory: accumulated evidence of conscious and unconscious bolstering of the status quo. *Polit. Psychol.* 25 881–919. 10.1111/j.1467-9221.2004.00402.x

[B24] Jost J. T. Kay A. C. (2005). Exposure to benevolent sexism and complementary gender stereotypes: consequences for specific and diffuse forms of system justification. *J. Pers. Soc. Psychol.* 88 498–509. 10.1037/0022-3514.88.3.498 15740442

[B25] Jost J. T. Ledgerwood A. Hardin C. D. (2008a). Shared reality, system justification, and the relational basis of ideological beliefs. *Soc. Personal. Psychol. Compass* 2 171–186. 10.1111/j.1751-9004.2007.00056.x

[B26] Jost J. T. Pelham B. W. Carvallo M. R. (2002). Non-conscious forms of system justification: implicit and behavioral preferences for higher status groups. *J. Exp. Soc. Psychol.* 38 586–602. 10.1016/S0022-1031(02)00505-X

[B27] Jost J. T. Pelham B. W. Sheldon O. Ni Sullivan B. (2003). Social inequality and the reduction of ideological dissonance on behalf of the system: evidence of enhanced system justification among the disadvantaged. *Eur. J. Soc. Psychol.* 33 13–36. 10.1002/ejsp.127

[B28] Jost J. T. Rudman L. A. Blair I. V. Carney D. R. Dasgupta N. Glaser J. (2009). The existence of implicit bias is beyond reasonable doubt: a refutation of ideological and methodological objections and executive summary of ten studies that no manager should ignore. *Res. Organ. Behav.* 29 39–69. 10.1016/j.riob.2009.10.001

[B29] Jost J. T. van der Toorn J. (2012). “System justification theory,” in *Handbook of Theories of Social Psychology* Vol. 2 eds van Lange P. A. M. Kruglanski A. W. Higgins E. T. (London: Sage), 313–343.

[B30] Jost J. T. Wakslak C. J. Tyler T. R. (2008b). System justification theory and the alleviation of emotional distress: palliative effects of ideology in an arbitrary social hierarchy and in society. *Adv. Group Process* 25 181–211. 10.1016/S0882-6145(08)25012-5

[B31] Kay A. C. Jost J. T. (2003). Complementary justice: effects of “poor but happy” and” poor but honest” stereotype exemplars on system justification and implicit activation of the justice motive. *J. Pers. Soc. Psychol.* 85 823–837. 10.1037/0022-3514.85.5.823 14599247

[B32] Kay A. C. Jost J. T. Young S. (2005). Victim derogation and victim enhancement as alternate routes to system justification. *Psychol. Sci.* 16 240–246. 10.1111/j.0956-7976.2005.00810.x 15733206

[B33] Ko K. Weng C. (2011). Critical review of conceptual definitions of chinese corruption: a formal–legal perspective. *J. Contemp. China* 20 359–378. 10.1080/10670564.2011.565170

[B34] Kuang L. Liu L. (2012). Discrimination against rural-to-urban migrants: the role of the hukou system in China. *PLoS ONE* 7:e46932 10.1371/journal.pone.0046932 PMC3489849 23144794

[B35] Lambsdorff J. G. (2006). “Measuring corruption: the validity and precision of subjective indicators,” in *Measuring Corruption* eds Sampford C. Shacklock A. Connors C. Galtun F. (Aldershot: Ashgate), 81–100.

[B36] Ledgerwood A. Mandisodza A. N. Jost J. T. Pohl M. J. (2011). Working for the system: motivated defense of meritocratic beliefs. *Soc. Cogn.* 29 322–340. 10.1521/soco.2011.29.3.322

[B37] Leong C.-H. Lin W. (2009). “Show me the money! construct and predictive validation of the intercultural business corruptibility scale (IBCS),” in *Intercultural Relations in Asia* eds Leong C.-H. Berry J. W. (Singapore: World Scientific).

[B38] Li L. (2011). Performing bribery in China: guanxi-practice, corruption with a human face. *J. Contemp. China* 20 1–20. 10.1080/10670564.2011.520841

[B39] Li S. Triandis H. C. Yu Y. (2006). Cultural orientation and corruption. *Ethics Behav.* 16 199–215. 10.1207/s15327019eb1603_2

[B40] Mauro P. (1995). Corruption and growth. *Q. J. Econ.* 110 681–712. 10.2307/2946696

[B41] Mazar N. Aggarwal P. (2011). Greasing the palm can collectivism promote bribery? *Psychol. Sci.* 22 843–848. 10.1177/0956797611412389 21685379

[B42] Metlay D. (1999). “Institutional trust and confidence: a journey into a conceptual quagmire,” in *Social Trust and the Management of Risk* eds Cvetkovich G. Löfstedt R. (London: Earthscan), 100–116.

[B43] Mishler W. Rose R. (2001). What are the origins of political trust? Testing institutional and cultural theories in post-communist societies. *Comp. Polit. Stud.* 34 30–62.

[B44] Mishler W. Rose R. (2005). What are the political consequences of trust? A test of cultural and institutional theories in Russia. *Comp. Polit. Stud.* 38 1050–1078.

[B45] Morris S. D. Klesner J. L. (2010). Corruption and trust: theoretical considerations and evidence from Mexico. *Comp. Polit. Stud.* 43 1258–1285. 10.1177/0010414010369072

[B46] Ntayi J. M. Ngoboka P. Kakooza C. S. (2013). Moral schemas and corruption in Ugandan public procurement. *J. Bus. Ethics* 112 417–436. 10.1007/s10551-012-1269-7

[B47] Pavlou P. A. Tan Y.-H. Gefen D. (2003). “The transitional role of institutional trust in online interorganizational relationships,” in *Proceedings of the 36th Annual Hawaii International Conference on System Sciences*, Kohala Coast, HI.

[B48] Pellegrini L. (2011). “The effect of corruption on growth and its transmission channels” in *Corruption, Development and the Environment* ed Pellegrini L. (Netherlands: Springer), 53–74.

[B49] Phelan J. E. Rudman L. A. (2011). System justification beliefs, affirmative action, and resistance to equal opportunity organizations. *Soc. Cogn.* 29 376–390. 10.1521/soco.2011.29.3.376

[B50] Preacher K. J. Hayes A. F. (2008). Asymptotic and resampling strategies for assessing and comparing indirect effects in multiple mediator models. *Behav. Res. Methods* 40 879–891. 10.3758/BRM.40.3.879 18697684

[B51] Rabl T. (2011). The impact of situational influences on corruption in organizations. *J. Bus. Ethics* 100 85–101. 10.1007/s10551-011-0768-2

[B52] Rankin L. E. Jost J. T. Wakslak C. J. (2009). System justification and the meaning of life: are the existential benefits of ideology distributed unequally across racial groups? *Soc. Justice Res.* 22 312–333. 10.1007/s11211-009-0100-9

[B53] Rosenblatt V. (2012). Hierarchies, power inequalities, and organizational corruption. *J. Bus. Ethics* 111 237–251.

[B54] Rotberg R. (2009). Transnational corruption threatens global security. *Paper Presented at the World Economic Forum. Global Agenda Council on Corruption. Raising Our Game: Next Steps for Business, Government and Civil Society to Fight Corruption.* Geneva: World Economic Forum.

[B55] Rus A. Iglič H. (2005). Trust, Governance and Performance The Role of Institutional and Interpersonal Trust in SME Development. *Int. Sociol.* 20 371–391.

[B56] Sardžoska E. G. Tang T. L.-P. (2012). Work-related behavioral intentions in Macedonia: Coping strategies, work environment, love of money, job satisfaction, and demographic variables. *J. Bus. Ethics* 108 373–391. 10.1007/s10551-011-1096-2

[B57] Serra D. Wantchekon L. (2012). *New Advances in Experimental Research on Corruption.* Bingley: Emerald Group Publishing.

[B58] Tan X. Liu L. Huang Z. Zhao X. Zheng W. (2016a). The dampening effect of social dominance orientation on awareness of corruption: moral outrage as a mediator. *Soc. Indic. Res.* 125 89–102. 10.1007/s11205-014-0838-9

[B59] Tan X. Liu L. Huang Z. Zheng W. (in press) Working for the hierarchical system: the role of meritocratic ideology in the endorsement of corruption. *Polit. Psychol.* 10.1111/pops.12341

[B60] Tan X. Liu L. Zheng W. Huang Z. (2016b). Effects of social dominance orientation and right-wing authoritarianism on corrupt intention: The role of moral outrage. *Int. J. Psychol.* 51 213–219. 10.1002/ijop.12148 25683842

[B61] Transparency International (2014). *Corruption Perceptions Index 2014.* Available at: http://www.transparency.org/cpi2014/results

[B62] Treisman D. (2000). The causes of corruption: a cross-national study. *J. Public Econ.* 76 399–457. 10.1016/S0047-2727(99)00092-4

[B63] Treisman D. (2007). What have we learned about the causes of corruption from ten years of cross-national empirical research? *Annu. Rev. Polit. Sci.* 10 211–244. 10.1146/annurev.polisci.10.081205.095418

[B64] Vainio A. Mäkiniemi J.-P. Paloniemi R. (2014). System justification and the perception of food risks. *Group Process. Intergroup Relat.* 17 509–523. 10.1177/1368430213503502

[B65] van der Toorn J. Berkics M. Jost J. T. (2010). System justification, satisfaction, and perceptions of fairness and typicality at work: a cross-system comparison involving the US and Hungary. *Soc. Justice Res.* 23 189–210. 10.1007/s11211-010-0116-1

[B66] Wei S. (2000). How taxing is corruption on international investors? *Rev. Econ. Stat.* 82 1–11. 10.1162/003465300558533

[B67] Xin X. Rudel T. (2004). The context for political corruption: a cross-national analysis. *Soc. Sci. Q.* 85 294–309. 10.1111/j.0038-4941.2004.08502005.x

[B68] You J.-S. (2007). Corruption as Injustice. *Paper Presented at the Annual Meeting of the American Political Science Association* Chicago.

