# Supplementary material for: The Effects of General System Justification on Corruption Perception and Intent
Source: Front Psychol. 2016 Jul 26;7:1107. doi: 10.3389/fpsyg.2016.01107 (PMC4960914; doi:10.3389/fpsyg.2016.01107)
Supplement: Supplementary file 1 [file Data_Sheet_1.DOCX]

**Appendix A. General system justification scale**

1. In general, China is just and fair.
2. In general, Chinese society operates as it should.
3. China needs to be restructured (reverse-scored).
4. China is the best country in the world.
5. China serves the greatest good for its citizens.
6. Everyone in China has a fair shot at wealth and happiness.
7. China is getting worse every year (reverse-scored).
8. China is set up so that people usually get what they deserve.

**Appendix B. Corruption Perception Measure in Study 1**

1. Corruption always exists in different areas.

2. Most people who have opportunities to be corrupt will be corrupt.

3. At present, corruption is a very common phenomenon and has spread to almost every industry.

4. The problem of corruption is very severe in Chinese society today.

5. The phenomenon of corruption has appeared in universities and is becoming more and more serious.

**Appendix C. Corrupt Intention Measure (scenario and items)**

The scenario is as follows:

*Imagine that you have a friend (A) who wishes to pursue his studies abroad. A charitable organization, X, has offered an overseas scholarship. It only offers five places every year, and more than 50 candidates apply for those positions. Therefore, the selection will be based on the number of hours the applicant contributes to volunteer activities. The five candidates with the highest hours will receive a scholarship. You are the president of the volunteers association of your university. Your friend gives you ¥2000 and hopes that you will help him to receive a scholarship. Although he did not participate in any volunteer activities organized by your association, he wants you, as president of the volunteers association, to provide him with a false certification indicating that he has participated in many hours of volunteer activities. If you help your friend and give him the false certification, the probability that he will receive the scholarship will be greatly improved.*

1. I think that I will accept the money and give him false evidence.
2. I take the blame for my corrupt action (reverse-scored).
3. If I met the same situation again, I would still give the false certification.
4. If any of my friends were in this situation, I would help him/her to show my support.

**Appendix D. Manipulation of General System Justification**

The passage for the high general system justification condition:

*These days, many people in China feel disappointed with the nation’s condition. Many citizens feel that the country has reached a low point in terms of social, economic, and political factors.* *… It seems that many countries in the world are enjoying better social, economic, and political conditions than China. More and more Chinese express a willingness to leave China and emigrate to other nations.*

The passage for the low general system justification condition:

*These days, despite the difficulties the nation is facing, many people in China feel safer and more secure relative to the past. Many citizens feel that the country is relatively stable in terms of social, economic, and political factors.* *… It seems that compared with many countries in the world the social, economic, and political conditions in China are relatively good. Very few Chinese express a willingness to leave and emigrate to other nations.*

**Appendix E. Corruption Perception Measure in Studies 2 and 3**

1. It is acceptable for someone to accept a bribe in the course of their duties. 1: Agree strongly – 9: Disagree strongly.

2. In the last 5 years, how often have you or a member of your immediate family come across a public official who hinted they wanted or asked for a bribe, or favor in return for a service? 1: Never – 9: Always.

3. Where would you place your image of the world on the following scale: 1: Basically good – 9: Perverse and corrupt.

4. To get all the way to the top in China today, you have to be corrupt. 1: Agree strongly – 9: Disagree strongly.

5. How widespread do you think corruption is in the public service in China? 1: Hardly anyone is involved – 9: Almost everyone is involved.

6. In your opinion, how many politicians in China are involved in corruption? Would you say 1: Almost none – 9: Almost all.

7. In your opinion, how many government administrators in China are involved in corruption? 1: Almost none – 9: Almost all.

**Appendix F. Corrupt intention measure in Studies 2 and 3**

1 Business corruption is inevitable.

2 When dealing with a business partner, it is important to inform the authorities if the partner asks for a bribe.

3 A business negotiation should observe a standard form of ethics.

4 In some situations, it is acceptable to pay someone extra in order to get things done quickly, even if the law forbids such practices.

5 I would not compromise my moral and business ethics for the sake of increasing business sales.

6 Giving expensive gifts to business clients and partners is an acceptable form of practice in some situations.

7 When doing business, it is important to endorse the norms practiced by your business partners even if you do not agree with them.

8 The rules governing ethical business transactions change from one situation to another.

9 All business partners and clients should be treated the same way.

10 One should observe a strict set of business ethics even if your partners embrace different business values.

11 When doing business with some people, it is alright to offer a bribe to the person, if the person is known to be open to such practice.

12 It is important to blend in with the organization that you do business with, even if it means being biased in offering contracts.

13 There should be a standard set of business ethics and protocols in all situations.

14 Moral and ethical standards should be observed at all times when doing business with people who are new to you.
